# Venetoclax plus hypomethylating agents in newly diagnosed acute myeloid leukemia patients with *RUNX1::RUNX1T1*: a retrospective propensity score matching study

**DOI:** 10.1038/s41408-023-00948-x

**Published:** 2023-11-27

**Authors:** Miao Wang, Han-Yu Cao, Kai-Wen Tan, Qiao-Cheng Qiu, Yuan-Hong Huang, Shuai-shuai Ge, Zi-Hao Wang, Jia Chen, Xiao-Wen Tang, De-Pei Wu, Sheng-Li Xue, Zheng Li, Hai-Ping Dai

**Affiliations:** 1 National Clinical Research Center for Hematologic Diseases, Jiangsu Institute of Hematology, The First Affiliated Hospital of Soochow University, Suzhou, China; 2 Institute of Blood and Marrow Transplantation, Collaborative Innovation Center of Hematology, Soochow University, Suzhou, China

**Keywords:** Chemotherapy, Acute myeloid leukaemia

## To the Editor:

T(8;21)(q22;q22.1)/*RUNX1::RUNX1T1* is found in 5–10% of all AML cases and responds well to conventional chemotherapy [1]. The complete remission (CR) rate of *RUNX1::RUNX1T1* positive AML with standard 7 + 3 regimen is 78–88%, with a 5-year survival rate of 95% when treated with gemtuzumab ozogamicin in combination with high-dose cytarabine based induction and consolidation chemotherapy [2–4]. AML with *RUNX1::RUNX1T1* is classified as a favorable risk group in the guidelines [5].

Venetoclax (VEN) is a highly selective inhibitor of the anti-apoptotic protein BCL-2, acting by mimicking the BCL-2 homology domain 3. VEN in combination with azacitidine showed a composite complete remission (CRc) rate of 66.4% in untreated AML patients who were ineligible for intensive chemotherapy [6]. A phase 2 trial in our institute demonstrated a CRc rate of 78.3% for VEN plus decitabine in newly diagnosed young AML patients with adverse risk [7]. Because patients with *RUNX1::RUNX1T1* are sensitive to standard induction regimen with 7 + 3±gemtuzumab ozogamicin, most venetoclax-based clinical trials excluded *RUNX1::RUNX1T1* patients. For example, no patients with *RUXN1::RUNX1T1* were enrolled in the VIALE-A study [6]. Only 3 patients with favorable risk were enrolled in the VIALE-C study, but the cytogenetics were not described [8]. Therefore, there is a lack of data on the efficacy of VEN plus hypomethylating agent (VEN + HMA) in AML patients with *RUNX1::RUNX1T1*. Recently, a number of patients with *RUNX1::RUNX1T1* positive AML who were unfit for intensive chemotherapy received VEN + HMA induction treatment at our institute. Here, we retrospectively compared the efficacy of VEN + HMA with standard 7 + 3 in newly diagnosed *RUNX1::RUNX1T1* positive AML patients, using a propensity score-matched analysis.

**Fig. 1:**
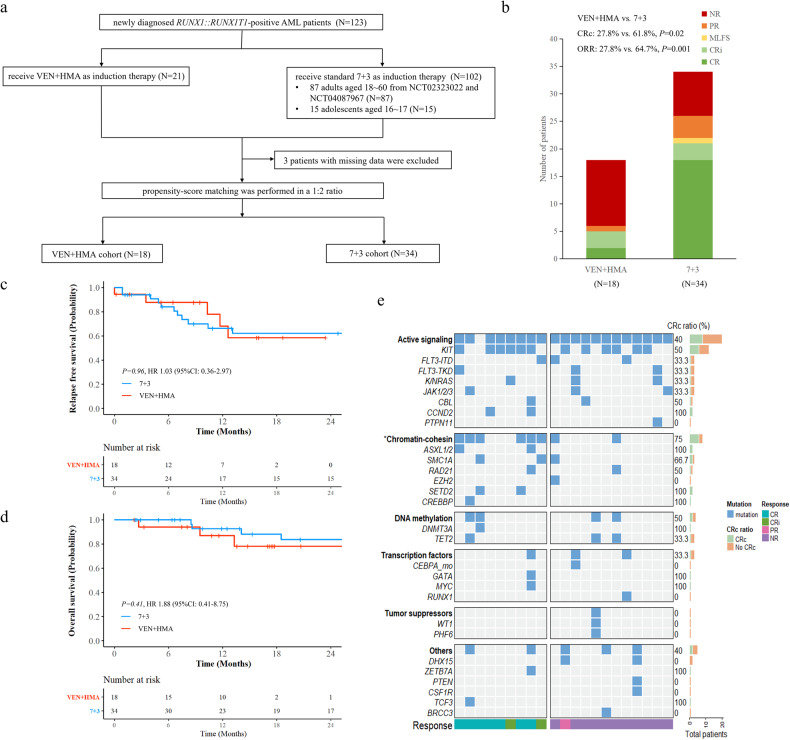
Study design, response outcomes, survival analysis, and mutational landscape of the unmatched VEN+HMA cohort. **a** Flow chart of inclusion of patients for propensity score matching analysis. **b** Treatment response of matched patients to the first course of induction treatment with VEN + HMA or standard 7 + 3. **c** Relapse-free survival in the matched cohorts. **d** Overall survival in the matched cohorts. **e** Mutational landscape of the unmatched 21 patients treated with VEN + HMA. Oncoprint shows mutational characteristics at diagnosis. Patients are grouped by best response after 1–2 induction cycles and are labeled with colored bars on the right. The filled bars on the right show the percentage of patients achieving CRc in the presence of each gene mutation. The asterisk indicates genes with a *P* < 0.05 percentage of CRc.

We collected data of 123 de novo AML patients with *RUNX1::RUNX1T1*, who received at least one course of induction therapy at the First Affiliated Hospital of Soochow University between April 2015 and February 2023. Twenty-one patients received induction with VEN + HMA, who were from clinical trial NCT04087967 and a real-world study (No.2022219). In total, 102 patients received induction with standard 7 + 3, including 87 adult patients from the NCT02323022 and NCT04087967 clinical trials, and 15 adolescents were included to match an unfit adolescent treated with VEN + HMA. Age, ECOG performance status, white blood cell count, bone marrow blasts, and *KIT* mutation status at diagnosis were selected for propensity score matching. Propensity scores were matched in a 1:2 ratio using the nearest-neighbor algorithm in the VEN + HMA versus standard 7 + 3 cohort. A standardized mean difference (SMD) threshold of less than 0.1 is considered to reduce bias between the two cohorts. After matching, 18 of 21 patients in the VEN + HMA cohort and 34 of 99 patients (3 were excluded because of missing data) in the 7 + 3 cohort were paired (Fig. 1a), with the SMD of 0.02 (Supplementary Fig. 1). There was no difference in the baseline and genetic characteristics between the matched cohorts (Table 1). The study was performed with approval from our institutional review committee (No. 2023248). All patients provided written informed consent.

**Table 1:** Baseline characteristics of patients in matched cohorts.

Variables	VEN + HMA (*N* = 18)	7 + 3 (*N* = 34)	*P* value
Age, years, median (range)	39.5 (17–69)	41.5 (16–59)	0.46
Male sex, *n* (%)	12 (66.7)	18 (52.9)	0.34
*ECOG performance status, n (%)*			0.73
0–1	13 (72.2)	23 (67.6)	
≥2	5 (27.8)	11 (32.4)	
*WBC at diagnosis, ×10*^*9*^*/L, n (%)*			0.54
> 10	9 (50.0)	14 (41.2)	
≤ 10	9 (50.0)	20 (58.8)	
*Platelet at diagnosis, ×10*^*9*^*/L, n (%)*			0.39
> 20	10 (55.6)	23 (67.6)	
≤ 20	8 (44.4)	11 (3.7)	
Bone marrow blasts (%), median (range)	57.3 (35.0–72.8)	52.0 (32.2–73.0)	0.72
*Karyotype, n (%)*			
Sole *t* (8;21)	6 (33.3)	12 (35.3)	0.75
*t* (8;21) with ACA	11 (61.1)	18 (52.9)	
Loss of X/Y	8 (72.7)	12 (66.7)	0.64
del(9q)	1 (9.1)	4 (22.2)	
Complex karyotype^a^	1 (9.1)	4 (22.2)	
del(7q)/−7	0	1 (5.6)	
Trisomy 8	0	1 (5.6)	
UK or failure	1 (5.6)	4 (11.8)	
*Co-mutation, n (%)*			
Signal genes	5 (27.8)	7 (20.6)	
*FLT3-ITD/TKD*	3 (16.7)	4 (11.8)	
*JAK1/2/3*	9 (50)	20 (58.8)	
*KIT*	3 (16.7)	6 (17.6)	
*NRAS/KRAS*	1 (5.6)	4 (11.8)	
Chromatin modification genes	3 (16.7)	3 (8.8)	
* ASXL1/2*	1 (5.6)	1 (5.6)	
*EZH2*	2 (11.1)	1 (5.6)	
*SETD2*	1 (5.6)	0	
*CREBBP*	2 (11.1)	2 (5.9)	
*DNA methylation genes*			
*DNMT3A*			
*TET2*	5 (27.8)	4 (11.8)	
Cohesin genes			0.17
*RAD21*	1 (5.6)	1 (2.9)	
*SMC1A/SMC3*	3 (16.8)	1 (2.9)	
Others			
*DHX15*	2 (11.1)	1 (2.9)	
*DNM2*	1 (11.1)	2 (5.9)	
*TP53*	0	0	
*ZBTB7A*	1 (5.6)	2 (5.9)	
*Immunophenotype, n/N (%)*			
CD19 expression	14/18 (77.8)	24/34 (70.6)	0.58
CD56 expression	11/18 (61.1)	15/22 (68.2)	0.64
*Extramedullary disease, n (%)*			0.23
Yes	2 (11.1)	1 (2.9)	
No	16 (88.9)	33 (97.1)	
Allo-HSCT			0.89
Yes	6 (44.4)	12 (35.3)	
No	12 (66.6)	22 (64.7)	

^a^A complex karyotype was defined as the presence t(8;21) and 2 or more other abnormalities.

Abbreviations: *ACA* additional cytogenetic abnormalities, *Allo-HSCT* allogeneic hematopoietic stem cell transplantation, *ECOG* Eastern Co-operative Oncology Group, *UK* unknown.

*RUNX1::RUNX1T1* was detected and quantified with real-time quantitative RT-PCR (qPCR), with a positive threshold defined as ≥2%. Multiparameter flow cytometry (MFC) was performed to detect measurable residual disease (MRD), with a positive threshold of 0.1% [5]. CRc is defined as CR plus CR with incomplete blood count recovery (CRi). Overall response rate (ORR) is defined as CRc plus morphologic leukemia-free state. Overall survival (OS) is defined as the time from diagnosis to death of any cause or censored at the last follow-up. Relapse-free survival (RFS) is defined as the time from the achievement of CRc to the time of relapse or death from any cause, whichever occurs first. For exploratory analysis, the chromatin-modifying genes *ASXL1/2*, *BCOR*, *EZH2*, *SETD2*, and cohesin complex-related genes *RAD21*, *SMC1A*, *SMC3*, and *STAG2* were defined as a set of genes named chromatin-cohesin gene in this study.

Patients in the VEN + HMA cohort received azacytidine (AZA, 75 mg/m^2^, on days 1–7) or decitabine (20 mg/m^2^, on days 1–5), in combination with VEN began at 100 mg on day 1 and increased stepwise over 3 days to reach the target dose of 400 mg on days 3–28. Dose adjustments for concomitant VEN with CYP3A4 inhibitors were made according to the VIALE-A study [6]. Patients who achieved CRc received at least 2 cycles of consolidation therapy with high-dose cytarabine or VEN + HMA (for older or unfit patients). Patients in partial remission (PR) or no response (NR) were treated with re-induction regimens at physicians’ discretion.

In unmatched patients, after one course of induction treatment, the ORR of the VEN + HMA cohort was significantly lower than that of the standard 7 + 3 cohort (38.1% vs. 74.5%, *P* = 0.001). The CRC rate in the VEN + HMA cohort was also significantly lower than that of the standard 7 + 3 cohorts (33.3% vs. 73.5%, *P* < 0.001) (Supplementary Fig. 2).

In matched patients, the ORR and CRc rate after the first course of induction treatment remained significantly lower in the VEN + HMA cohort compared to the standard 7 + 3 cohorts (27.8% vs. 64.7%, *P* = 0.01 for ORR, and 27.8 % vs. 61.8%, *P* = 0.02 for CRc rate) (Fig. 1b, (Supplementary Table 1). In the matched VEN + HMA cohort, 13 patients failed the first cycle of induction. Of whom, 4 patients received a second cycle of VEN + HMA. However, only one patient achieved CRi, and 3 patients still had no response. Two patients switched to standard 7 + 3 and both achieved CR. In the matched 7 + 3 cohort, 12 patients failed the first induction treatment. Two of whom received a second cycle of standard 7 + 3, one achieved CR and one had PR. None of them switched to the VEN + HMA regimen.

We further compared the remission depth in responders (who attained CRc after 1–2 cycles of induction) of each cohort at the time of completion of induction and the second consolidation. Six of the 18 patients in the VEN + HMA cohort achieved CR/CRi, and 5/6 had qPCR MRD data. Twenty-two of the 34 patients in the 7 + 3 cohort achieved CR/CRi, and 18/22 had qPCR MRD data. There was no difference in the MFC MRD-negative rate (60% vs. 66.7%, *P* = 1.00) (Supplementary Table 1) or the qPCR MRD-negative CR rate (100% vs. 83.3%, *P* = 1.00) in the VEN + HMA compared with standard 7 + 3 cohort, respectively (Supplementary Table 2).

At a median follow-up of 14.8 months for patients in the VEN + HMA cohort and 28.6 months for patients in the standard 7 + 3 cohort, median OS was not achieved in either cohort. There was no difference in the 2-year RFS (58.5% vs. 62.2%, *P* = 0.96) and 2-year OS (78.2% vs. 83.5%, *P* = 0.41) between the two cohorts (Fig. 1c, d). Six patients in the matched VEN + HMA cohort underwent allo-HSCT. When patients were censored at allo-HSCT, the 2-year RFS and 2-year OS also did not differ between the cohorts (42.9% vs. 51.9%, *P* = 0.93 for RFS, 78.2% vs. 83.5%, *P* = 0.51 for OS) (Supplementary Fig. 3a, b). Subgroup analyses of the VEN + HMA cohort showed no difference in 2-year RFS (not reached, *P* = 0.85) and 2-year OS (not reached, *P* = 0.26) between responders and non-responders (Supplementary Fig. 3c, d).

To investigate the factors influencing the sensitivity of AML patients with *RUNX1::RUNX1T1* to VEN + HMA, we analyzed data from the 21 unmatched patients treated with VEN + HMA. Genetic profiles of responders and non-responders were depicted in Fig. 1e. Nine of 21 (42.9%) patients who achieved CR/CRi within 2 cycles of the VEN + HMA regimen were categorized as responders (7 and 2 patients responded after cycle 1 and cycle 2, respectively), whereas the other 12 patients were categorized as non-responders. There were no differences in clinical characteristics between responders and non-responders. However, chromatin-cohesin gene mutations, including *ASXL1/2*, *BCOR*, *EZH2*, *STAG2*, *RAD21*, *SMC1A*, *SMC3, KMT2A*, *SETD2*, were more frequently detected in responders (6/9, 66.7%) compared to the non-responders (2/12, 16.7%) (*P* = 0.03) (Supplementary Table 3). These data suggest that responders of AML patients with *RUNX1::RUNX1T1* in the VEN + HMA cohort were significantly enriched for mutations relating to chromatin-cohesin genes. This finding is consistent with a recent report that mutations in chromatin-cohesin genes predicted a better response to VEN-based therapies [9].

In 4 retrospective studies from China, data showed only 3 of 9 (33.3%) newly diagnosed and none of 16 relapsed/refractory *RUNX1::RUNX1T1* positive AML patients responded to VEN + AZA [9–12] (Supplementary Table 4). Our results are in line with these studies. According to the literature, we suggested that the mechanisms of a relative inferior response to VEN + HMA in *RUNX1::RUNX1T1* positive AML include: 1. *BCL-2* gene expression was low in patients with *RUNX1::RUNX1T1* in a multicenter study [13], which resulted in the insensitivity of VEN in these patients. 2. BCL-XL expression is upregulated in *RUNX1::RUNX1T1* positive cells, which plays an essential role in resistance to VEN [14] 3. Active signaling genes including *KIT*, *FLT3-*ITD, and *NRAS/KRAS* are frequently co-mutated with *RUNX1::RUNX1T1*, which were identified as predictors of inferior response to VEN + HMA in AML patients [9, 15].

In conclusion, our results showed that patients with *RUNX1::RUNX1T1* positive AML responded suboptimally to VEN + HMA than to the standard 7 + 3 regimen after one course of induction treatment. Co-mutations in the chromatin-cohesin genes may facilitate the identification of *RUNX1::RUNX1T1* positive AML patients who respond to VEN + HMA. This study provides clues for the selection of induction regimens for *RUNX1::RUNX1T1* positive AML patients. Because of the limited sample size and the fact that many non-responders switched to other regimens after the first cycle of VEN + HMA, the results of this study warrant an investigation in a prospective randomized controlled study.

### Supplementary information

SUPPLEMENTAL MATERIAL

## Data Availability

The data that support the findings of this study are available from the corresponding author upon reasonable request.
