# Supplementary material for: Venetoclax plus hypomethylating agents in newly diagnosed acute myeloid leukemia patients with RUNX1::RUNX1T1: a retrospective propensity score matching study
Source: Blood Cancer J. 2023 Nov 27;13(1):173. doi: 10.1038/s41408-023-00948-x (PMC10682468; doi:10.1038/s41408-023-00948-x)
Supplement: Supplementary file 1 — SUPPLEMENTAL MATERIAL [file 41408_2023_948_MOESM1_ESM.pdf]

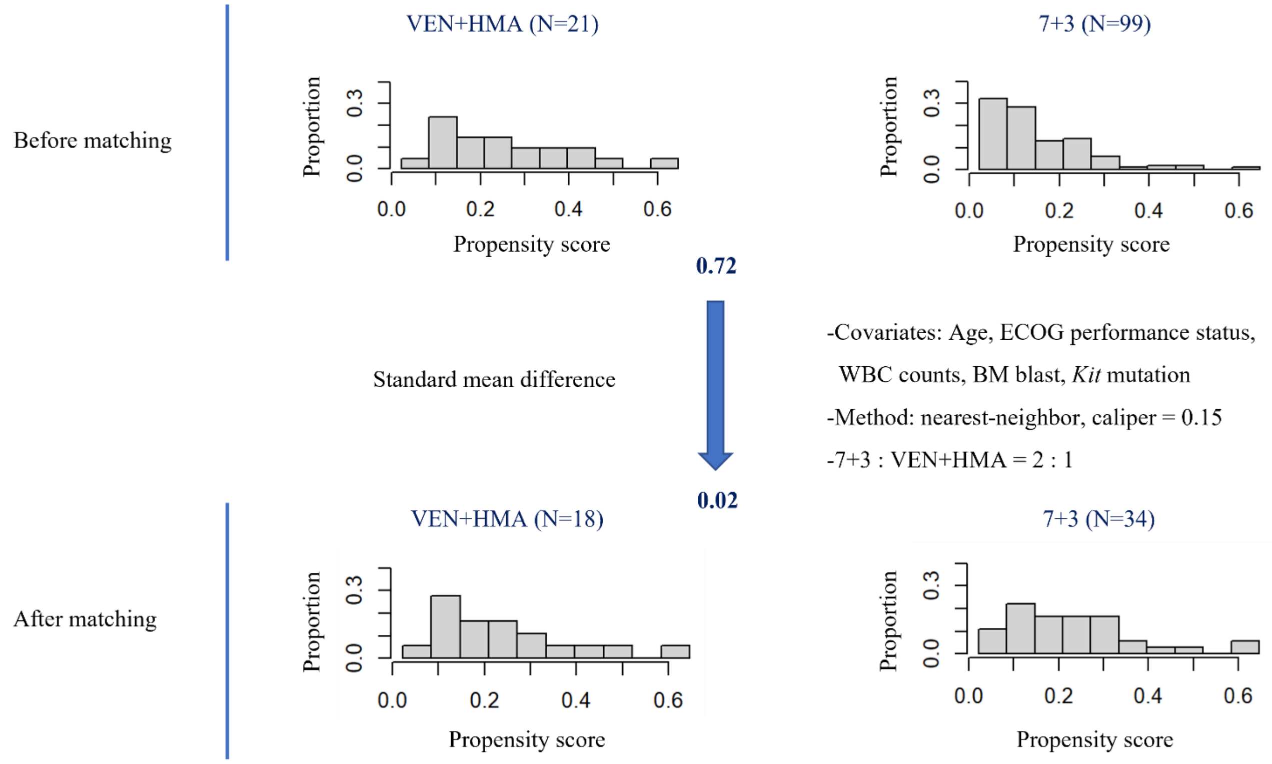

Figure S1. Propensity score matching process.
Abbreviations: BM, bone marrow; ECOG, Eastern Co-operative Oncology Group; HMA, hypomethylating agent; VEN, venetoclax, WBC, white blood cell.

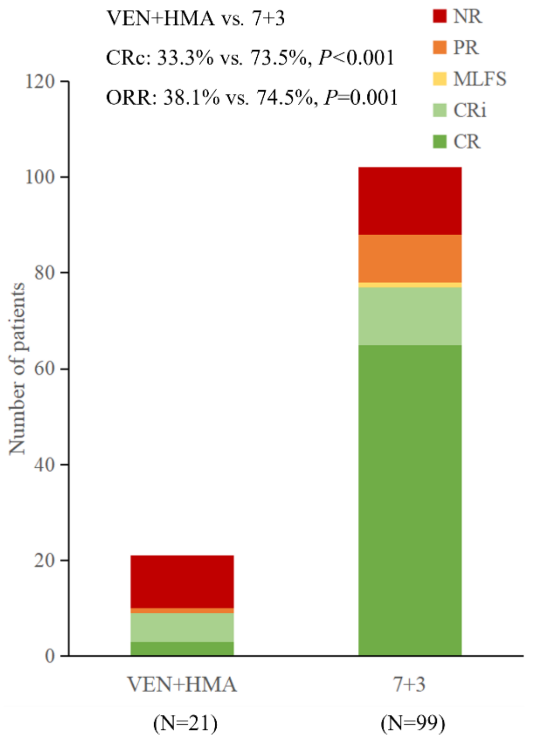

Figure S2. Response outcomes after cycle 1 in unmatched cohorts. Abbreviations: CR, complete remission, CRi, complete remission with incomplete hematologic recovery, HMA, hypomethylating agents, NR, no response, PR, partial remission, VEN, venetoclax.

a b c d

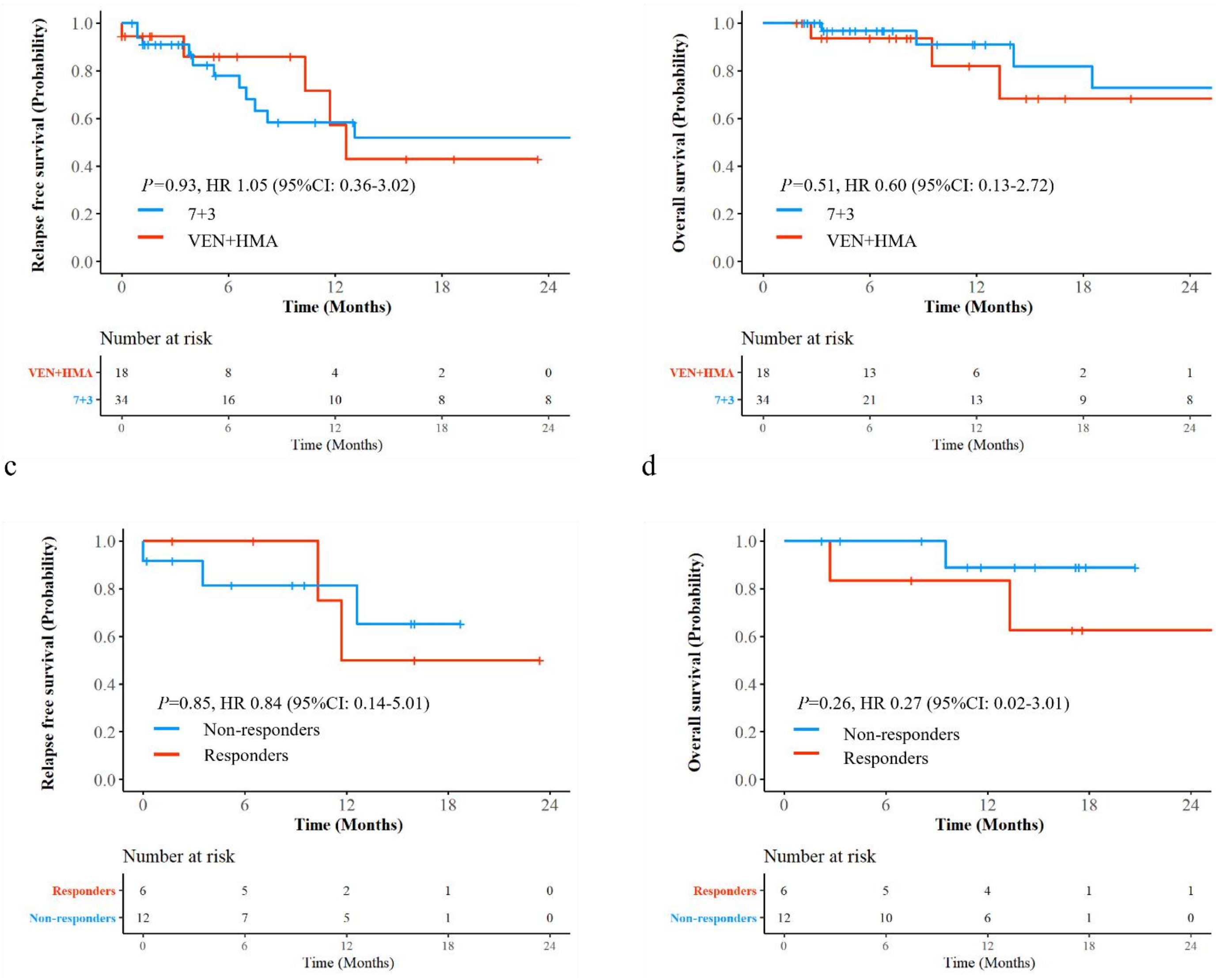

Figure S3. (a) Relapse-free survival and (b) overall survival when censored at allo-HSCT in the matched cohorts. Subgroup analysis of (c) Relapse-free survival and (d) overall survival between responders and non-responders in the matched VEN+HMA cohort.

Supplementary Table 1. Comparison of treatment responses in matched cohorts.

	VEN+HMA (N=18)	Standard 7+3 (N=34)	*P* value
Response outcome after 1 cycle of induction			
**Overall response rate (ORR)**	5 (27.8)	22 (64.7)	0.01
CR	2 (11.1)	18 (53.0)	
CRi	3 (16.7)	3 (8.8)	
MLFS	0	1 (2.9)	
**PR**	1 (5.5)	4 (11.8)	
**NR**	12 (66.7)	8 (23.5)	
**Composite complete response rate (CRc)**	5 (27.8)	21 (61.8)	0.02
MFC MRD available	5	18	
CR with MFC MRD negative	3 (60.0)	12 (66.7)	1.00
CR with MFC MRD positive	2 (40.0)	6 (33.3)	
Response outcome after 1~2 cycles of induction			
**Overall response rate (ORR)**	6 (33.3)	23 (67.7)	
CR	2 (11.1)	19 (76.9)	
CRi	4 (22.2)	3(7.7)	
MLFS	0	1 (2.9)	
**PR**	1 (5.5)	3 (8.8)	
**NR**	11 (61.1)	8 (23.5)	
**Composite complete response rate (CRc)**	6 (33.3)	22 (64.7)	
MRD available	6	19	
CR with MRD negative	4 (66.7)	13 (68.4)	1.00
CR with MRD positive	2 (33.3)	6 (31.6)	

Abbreviations: CR: complete remission; CRi: CR with incomplete hematologic recovery ; MLFS； morphologic leukemia-free state; MRD: measurable residual disease; NR: no response； PR: partial remission.

Supplementary Table 2. Molecular MRD assessment after the 2nd consolidation in matched cohorts.

	VEN+HMA (n=5) §	Standard 7+3 (n=22) *	*P* value
qPCR MRD available	5	18	
qPCR MRD negative	5 (100.0)	15 (83.3)	1.00
qPCR MRD positive	0	3 (16.7)	
Relapse/Death from MRD negative	2/5(40)	2/15(13.3)	0.25

* 1 patient underwent allo-HSCT after standard 7+3 reinduction whose MRD (qPCR) was negative before HSCT, is included in this setting

§ 6 patients achieved CRc after 1~2 cycles of VEN+HMA as induction, 1 patient died from infection at first consolidation.

The ratio of *RUNX1/RUNX1T1* transcripts to ABL1 ≥2% is considered as qPCR MRD positive.

Supplementary Table 3. Exploratory analysis of baseline characteristics of patients in the unmatched VEN+HMA cohort.

Baseline characteristics	Responders (n=9)	Non-responders (n=12)	*P* value
Age, years, median (range)	48.0 (25-65)	37.5 (17-75)	0.40
Male sex, n (%)	6 (66.7)	8 (66.7)	1.00
ECOG performance status, n (%)			0.66
0~1	7 (77.8)	8 (66.7)	
≥ 2	2 (22.2)	4 (33.3)	
WBC at diagnosis, $\times 10^9$/L, n (%)			1.00
> 10	6 (66.7)	7 (58.3)	
≤ 10	3 (33.3)	5 (41.7)	
Platelet at diagnosis, $\times 10^9$/L, n (%)			1.00
> 20	5 (55.6)	6 (50.0)	
≤ 20	4 (44.4)	6 (50.0)	
Bone marrow blasts (%), median (range)	43.0 (35.8-59.8)	61.0 (37.6-78.3)	0.58
Karyotype, n/N (%)			0.60
t (8;21) or t (8;21) with loss of X/Y	7/8(87.5)	8/12(66.7)	
other abnormalities	1/8(12.5)	4/12(33.3)	
Co-mutation, n/N (%)			
Active signals			
CBL	1/9 (11.1)	1/12 (8.3)	
CCND2	2/9 (22.2)	0	
FLT3-ITD	1/9 (11.1)	2/12 (16.7)	
FLT3-TKD	1/9 (11.1)	2/12 (16.7)	
JAK1/2/3	1/9 (11.1)	2/12 (16.7)	
KIT	6/9 (66.7)	6/12 (50.0)	
D816	2/9 (22.2)	6/12 (50.0)	
Others	4/9 (44.4)	0	
NRS/KRAS	1/9 (11.1)	2/12 (16.7)	
PTPN11	0	1/12 (8.3)	
Chromatin-cohesin*	6/9 (66.7)	2/12 (16.7)	**0.03**
DNA methylation			
DNMT3A	1/9 (11.1)	1/12 (8.3)	1.00
TET2	1/9 (11.1)	3/12 (25.0)	0.60
Others			
DHX15	0	2/12 (16.7)	
ZBTB7A	1/9 (11.1)	0	
Immunophenotype, n/N (%)			
CD19 expression	8/9 (88.9)	8/12 (66.7)	0.34
CD56 expression	6/9 (66.7)	8/12 (66.7)	1.00

Results reported as n (%), n/N (%), or median [range].

* chromatin-cohesin genes: *ASXL1/2, BCOR, EZH2, KMT2A SETD2, CREBBP, RAD21, SMC1A, SMC3, STAG2*

Supplementary Table 4. Efficacy of venetoclax combined with azacytidine in *RUNX1::RUNX1T1*-positive AML patients from the literature.

				newly diagnosed AML					relapsed or refractry AML				
Reference	Total Number	Regimen	Cycles	Response			Numbers	CRc rate	Response			Numbers	CRc rate
				CR/CRi	PR	NR			CR/CRi	PR	NR		
Yu, W.J.*et al.*[12]	7	VEN+AZA	1	2	UK	UK	4	50%	0	UK	UK	3	0%
Mi, R. H. *et al.*[10]	6	VEN+AZA	1	1	0	4	5	25%	0	0	1	1	0%
Weng, G.Y, *et al.*[9]	5	VEN+AZA	Median of 2 (range, 1–4)						0	1	4	5	0%
Yu, G.P, *et al.*[11]	7	VEN+AZA	UK						0	UK	UK	7	0%

Abbreviations: AZA, azacytidine; ND, newly diagnosed; R/R, relapsed or refractory; UK, unknown; VEN, venetoclax.

Supplementary Table 5. Data of patients in matched VEN+HMA cohort who underwent allo-HSCT.

**Pt No.**	**Sex**	**Age**	**Donor type**	**Reasons for allo-HSCT**	**Copies of *RUNX1::RUNX1T1* by qPCR**		**Current status**
					**Before allo-HSCT**	**last follow-up**	
3	F	17	HID	*KIT* D816 mut relapse	1323%	0	MRD negative CR
13	F	44	MSD	*KIT* D816 mut Persistent MRD	0.47%	0.05%	MRD negative CR
14	F	30	MUD	*KIT* D816 mut EMD	0.1%	0	MRD negative CR
15	M	37	HID	MRD elevation>1 log after consolidation	69.89%	0	Relapse
17	M	41	MSD	*KIT* D816 mut relapse	147.24%	0.04%	MRD negative CR
21	M	32	MUD	*KIT* D816 mut relapse	8.91%	67.53%	relapse

Abbreviations: Allo-HSCT: allogeneic hematopoietic stem cell transplantation; CR: complete remission; EMD: extramedullary disease; F: female; HID: haplo-identical donor; M: male; MRD: measurable residual disease; MSD: matched sibling donor; MUD: matched unrelated donor; mut: mutation; Pt: patient.
